# Comparative Analysis of Fermentation Characteristics of *Saccharomyces cerevisiae* and Non-*Saccharomyces* Yeast Strains Isolated from Nuruk and Makgeolli

**DOI:** 10.3390/microorganisms14061352

**Published:** 2026-06-16

**Authors:** Jieun Kim, Jinju Park, So-Young Kim, Chan-Woo Kim

**Affiliations:** Fermented and Processed Food Research Division, Department of Food Sciences, National Institute of Crop and Food Science, Rural Development Administration, Wanju 55365, Republic of Korea

**Keywords:** *Saccharomyces cerevisiae*, non-*Saccharomyces* yeasts, volatile aroma compounds, organic acids, free amino acids

## Abstract

Traditional fermented foods harbor diverse microbial resources and provide an important reservoir for fermentation applications. We compared selected fermentation-related metabolite profiles of yeast strains isolated from traditional Korean fermentation starters and fermented foods, including nuruk and makgeolli. A total of 63 *Saccharomyces cerevisiae* strains and 23 non-*Saccharomyces* strains were cultured in YPD medium for 24 h, and volatile compounds, organic acids, and free amino acid-related compounds were analyzed. Non-*Saccharomyces* strains generally showed higher levels of ester compounds, whereas *S. cerevisiae* strains exhibited relatively higher levels of alcohol compounds. In particular, *Kluyveromyces marxianus* KJ-L showed high levels of both ester and alcohol-related volatile compounds, suggesting its potential as a candidate strain for further aroma-related evaluation. Organic acid and free amino-related profiles also differed between the two yeast groups. These results highlight the distinct metabolic traits of *S. cerevisiae* and non-*Saccharomyces* strains and provide useful screening information for selecting candidate microbial resources for further validation in fermented food systems.

## 1. Introduction

Fermentation is a microbially driven process widely used in the production of fermented foods, and its application predates the scientific understanding of microorganisms. Traditional fermented foods harbor diverse microbial resources, and securing indigenous fermentative microorganisms is essential for the development of traditional Korean fermented foods [1]. Nuruk is a traditional Korean cereal-based fermentation starter containing diverse microorganisms and enzymes, and makgeolli is a traditional Korean rice wine whose quality is strongly influenced by the microbial composition and metabolic activity of nuruk.

In alcoholic beverage production, microorganisms convert raw materials into various metabolites including ethanol, organic acids, and aromatic compounds, which collectively determine product quality [2]. In particular, organic acids can react with alcohols during fermentation to form esters, contributing to the diverse aroma profiles of fermented beverages [3,4]. Therefore, the composition of volatile aroma compounds may vary depending on the metabolic activities of the microorganisms involved.

Yeasts play a key role in producing these compounds and shaping the overall quality of alcoholic beverages [5]. *Saccharomyces cerevisiae* is the most widely used yeast in alcoholic fermentation due to its tolerance to ethanol, sugar, and low-pH conditions, as well as its efficient fermentation performance. It rapidly converts sugars into ethanol while producing various aromatic compounds and inhibiting undesirable microorganisms, making it the dominant species in industrial fermentation processes [6,7,8].

Various yeast strains, including *S. cerevisiae* and non-*Saccharomyces* species, have been isolated from traditional Korean fermentation starters and foods, including nuruk and makgeolli. However, despite this microbial diversity, most industrial fermentation processes primarily rely on *S. cerevisiae*, and many non-*Saccharomyces* yeasts have not been commercially utilized. In Korea, fermentation industries largely rely on imported *S. cerevisiae* strains, highlighting the need to secure competitive indigenous yeast resources [9].

Recent studies have demonstrated that non-*Saccharomyces* yeasts possess beneficial fermentation properties, including enhanced production of aromatic compounds and diverse enzyme activities [10,11]. These functional traits have been increasingly recognized as important contributors to flavor development in fermented foods. In particular, mixed fermentations involving non-Saccharomyces yeasts and *S. cerevisiae* have been shown to improve product quality and generate more complex aroma profiles [12,13,14]. Despite these advances, systematic comparative studies on the fermentation characteristics of *S. cerevisiae* and non-*Saccharomyces* strains isolated from traditional Korean fermented foods remain limited.

Therefore, in this study, 63 *S. cerevisiae* strains and 23 non-*Saccharomyces* strains isolated from traditional Korean fermentation starters and foods were cultivated in YPD medium to compare their fermentation-related metabolite profiles. Volatile aroma compounds, organic acids, and free amino acids were analyzed to evaluate their metabolic profiles. This study aims to elucidate the functional diversity of indigenous yeast strains and explore their application potential to improve the quality of fermented alcoholic beverages and support the development of indigenous yeast resources for fermentation industries.

## 2. Materials and Methods

### 2.1. Strains and Culture Conditions

A total of 63 *Saccharomyces cerevisiae* strains and 23 non-*Saccharomyces* strains used in this study were isolated from Korean fermented starters and foods and selected by the National Institute of Crop and Food Science. All strains used in this study are listed in Table 1. Commercial yeast strains, *S. cerevisiae* Fermivin (DSM Food Specialties, Delft, The Netherlands), La Parisienne (Marcq-en-Baroeul Cedex, France), and the laboratory strain S288C, were used as reference strains. For inoculum preparation, each strain was cultured in YPD medium (10 g/L yeast extract, 20 g/L peptone, and 20 g/L dextrose) at 30 °C and 250 rpm for 24 h. The cells were then harvested by centrifugation at 10,000 rpm for 10 min, resuspended, and adjusted to an initial optical density of 0.5 at 600 nm (OD600). The adjusted cell suspensions were inoculated into 20 mL of YPD medium in a 100 mL Erlenmeyer flask for the main culture. Main cultures were conducted at 30 °C and 150 rpm for 24 h. YPD medium was used as a standardized culture medium to compare strain-dependent metabolite profiles under uniform nutrient conditions while minimizing variation caused by complex raw materials and microbial interactions. After cultivation, fermentation samples were collected and stored at −20 °C until analysis.

### 2.2. Volatile Aroma Compounds Analysis

Volatile aroma compounds in the fermented media were extracted using the stir bar sorptive extraction (SBSE) method. Samples were prepared by centrifugation at 10,000 rpm for 10 min. Then, 2.4 g of NaCl was dissolved in 10 mL of the supernatant, and 10 µL of 10,000 µg/mL methyl cinnamate in ethanol was added as an internal standard. The prepared samples were stirred at 800 rpm for 1 h using a PDMS (polydimethylsiloxane) stir bar (0.5 mm film thickness, 10 mm length; Gerstel, Mülheim an der Ruhr, Germany). After extraction, the stir bar was removed, rinsed with distilled water to eliminate impurities, and mounted in a thermal desorption system (TDS3, Gerstel, Germany) equipped with a cooled injection system (CIS4, Gerstel, Germany). The adsorbed compounds were thermally desorbed at 260 °C and subsequently analyzed by GC-MS/MS (Agilent Technologies, Santa Clara, CA, USA). A DB-WAX column (60 m × 0.32 mm i.d., 0.25 µm film thickness, Agilent Technologies, CA, USA) was used for separation, with helium as the carrier gas at a flow rate of 1.2 mL/min. The oven temperature was initially held at 40 °C for 5 min and then increased to 240 °C at a rate of 5 °C/min. Volatile compounds were semi-quantitatively estimated based on the peak area ratio of each compound to the internal standard. Therefore, the reported values should be interpreted as relative estimates rather than absolute concentration.

### 2.3. Analysis of Organic Acids

For analysis of organic acids, samples were centrifuged at 10,000 rpm for 10 min, and filtered through a 0.2 μm nylon membrane filter (Millipore Co., Tokyo, Japan). Organic acids were analyzed using HPLC (LC-20A, Shimadzu Co., Kyoto, Japan) equipped with two pumps and a TSKgel ODS-100 column (4.6 mm × 250 mm, Tosoh, Tokyo, Japan). An 8 mM perchloric acid solution was used as a mobile phase for pump A at a flow rate of 1.0 mL/min. The injection volume was 10 µL, and the column oven temperature was maintained at 40 °C. After separation, the eluate was mixed with a post-column reaction solution containing 0.2 mM bromothymol blue, 15 mM Na_2_HPO_4_, and 7 mM NaOH, delivered by pump B at a flow rate of 1.0 mL/min. Detection was performed using a UV detector at 440 nm.

### 2.4. Free Amino Acid Analysis

For free amino acid analysis, 5% trichloroacetic acid (TCA; Sigma-Aldrich Co., St. Louis, MO, USA) was added to 0.5 mL of each sample. The mixture was centrifuged at 10,000 rpm and 4 °C for 10 min, and the supernatant was collected and filtered through a 0.2 µm nylon membrane filter (Millipore Co., Burlington, MA, USA) prior to analysis. Free amino acids were analyzed using an amino acid analyzer (L-8900, Hitachi, Ltd., Tokyo, Japan) equipped with a PF#2666 column (4.6 mm × 60 mm, Hitachi, Ltd., Japan). The injection volume was 20 µL. The column oven and reactor temperature were set at 57 °C and 136 °C, respectively. Ninhydrin solution (Wako Pure Chemical Industries, Ltd., Osaka, Japan) was used for color development.

### 2.5. Statistical Analysis

All experiments were performed in triplicate, and the results are presented as mean ± standard deviation. Statistical significance was evaluated by one-way analysis of variance (ANOVA) followed by Duncan’s multiple range test at *p* < 0.05 using SPSS software (version 23.0; SPSS Inc., Armonk, NY, USA). Heatmap analysis of volatile aroma compounds was performed using MetaboAnalyst 6.0.

## 3. Results

### 3.1. Volatile Compounds

**Figure 1:**
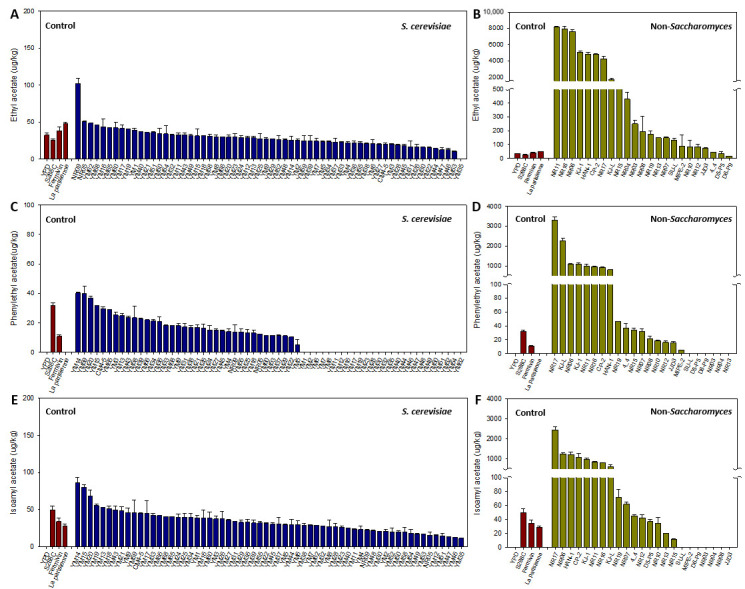
Volatile ester compounds in fermented media produced by different yeast strains and control yeasts. Ethyl acetate produced by *S. cerevisiae* strains (**A**) and non-*Saccharomyces* strains (**B**); phenylethyl acetate produced by *S. cerevisiae* strains (**C**) and non-*Saccharomyces* strains (**D**); isoamyl acetate produced by *S. cerevisiae* strains (**E**) and non-*Saccharomyces* strains (**F**).

**Figure 2:**
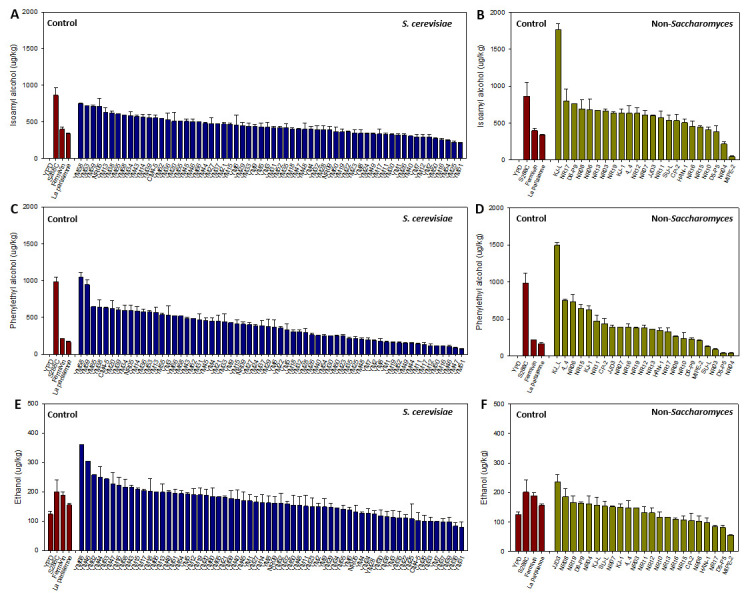
Volatile alcohol compounds in fermented media produced by different yeast strains and control yeasts. Isoamyl alcohol produced by *S. cerevisiae* strains (**A**) and non-*Saccharomyces* strains (**B**); phenylethyl alcohol produced by *S. cerevisiae* strains (**C**) and non-*Saccharomyces* strains (**D**); ethanol produced by *S. cerevisiae* strains (**E**) and non-*Saccharomyces* strains (**F**).

The selected volatile compounds detected in cultures of yeasts isolated from Korean foods and fermentation starters are shown in Figure 1 and Figure 2. A total of six compounds belonging to two chemical classes, esters and alcohols, were detected. The non-*Saccharomyces* strains showed significantly higher levels of selected ester compounds, particularly ethyl acetate and phenylethyl acetate than the *S. cerevisiae* strains (*p* < 0.05), whereas the *S. cerevisiae* strains showed significantly higher levels of alcohol compounds, especially isoamyl alcohol and phenylethyl alcohol (*p* < 0.05).

**Figure 3:**
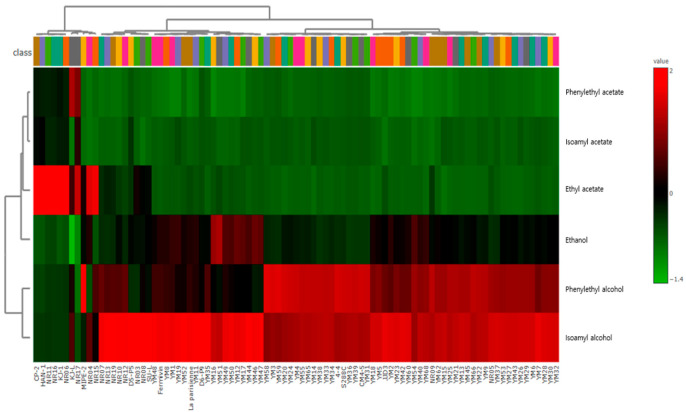
Clustering heatmap of volatile aroma compounds in fermented media produced by different *S. cerevisiae* and non-*Saccharomyces* strains, including control yeasts.

The major volatile compounds detected in the non-*Saccharomyces* group were ethyl acetate and phenylethyl acetate, which reached maximum concentrations of 8129.73 and 3292.71 μg/kg, respectively (Figure 1B,D). By contrast, the predominant compounds in *S. cerevisiae* were isoamyl alcohol and phenylethyl alcohol, with maximum concentrations of 749.53 and 1044.89 μg/kg, respectively (Figure 2A,C). Among non-*Saccharomyces* strains, *K. marxianus* KJ-L exhibited the highest production of both isoamyl alcohol and phenylethyl alcohol, reaching 1769.55 and 1493.13 μg/kg, respectively (Figure 2B,D). The non-*Saccharomyces* strains also showed a relatively diverse profile of selected ester compounds, including ethyl acetate, phenylethyl acetate, and isoamyl acetate (Figure 1). Among these strains, *W. anomalus* NR11 and NR16 produced the highest levels of ethyl acetate. Among the *S. cerevisiae* strains, YM14 produced the highest phenylethyl acetate concentration (40.23 μg/kg; Figure 1C), whereas much higher levels were observed in non-*Saccharomyces* strains, particularly *C. fabianii* NR17 and *K. marxianus* KJ-L (Figure 1D). *K. marxianus* KJ-L also produced the highest level of isoamyl alcohol among non-*Saccharomyces* strains (1769.55 μg/kg; Figure 2B). For phenylethyl alcohol, *K. marxianus* KJ-L showed the highest level (1493.13 μg/kg), followed by *S. fibuligera* 4-4 (Figure 2D). Among *S. cerevisiae* strains, YM58 and YM59 showed the highest production levels (Figure 2C). Heatmap analysis revealed a clear separation between *S. cerevisiae* and non-*Saccharomyces* groups (Figure 3). Alcohols were more abundant in *S. cerevisiae*, whereas esters were enriched in non-*Saccharomyces*.

### 3.2. Organic Acids

**Figure 4:**
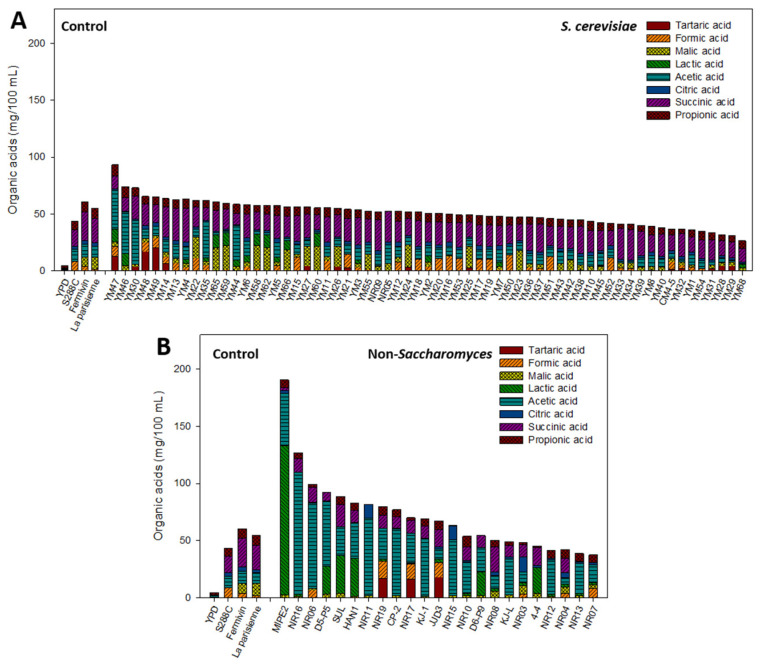
Concentrations of organic acids in fermented media produced by different yeast strains and control yeasts: (**A**) *S. cerevisiae* strains and (**B**) non-*Saccharomyces* strains.

Organic acid profiles are shown in Figure 4. Organic acid profiles differed significantly among strains and between yeast groups (*p* < 0.05). In *S. cerevisiae*, succinic acid and acetic acid were the predominant organic acids, whereas non-*Saccharomyces* strains showed higher production of acetic acid and lactic acid. The highest total organic acid production was observed in *S. cerevisiae* YM47 and *P. deserticola* MIPE2. Succinic acid ranged from 10.01 to 29.27 mg/100 mL in *S. cerevisiae* and from 0.00 to 27.44 mg/100 mL in non-*Saccharomyces* strains. Acetic acid ranged from 0.89 to 37.83 mg/100 mL in *S. cerevisiae* and from 0.00 to 106.27 mg/100 mL in non-*Saccharomyces* strains, with *W. anomalus* NR16 showing the highest production. Lactic acid production differed markedly between the two groups. YM47 showed the highest level among *S. cerevisiae* strains, whereas *P. deserticola* MIPE2 showed the highest production among non-*Saccharomyces* strains. Malic acid was highest in *S. cerevisiae* YM60, while *P. deserticola* NR03 showed the highest level among non-*Saccharomyces* strains. Citric acid was highest in *S. cerevisiae* YM12 and *P. deserticola* NR03 among non-*Saccharomyces* strains.

### 3.3. Free Amino Acids

**Figure 5:**
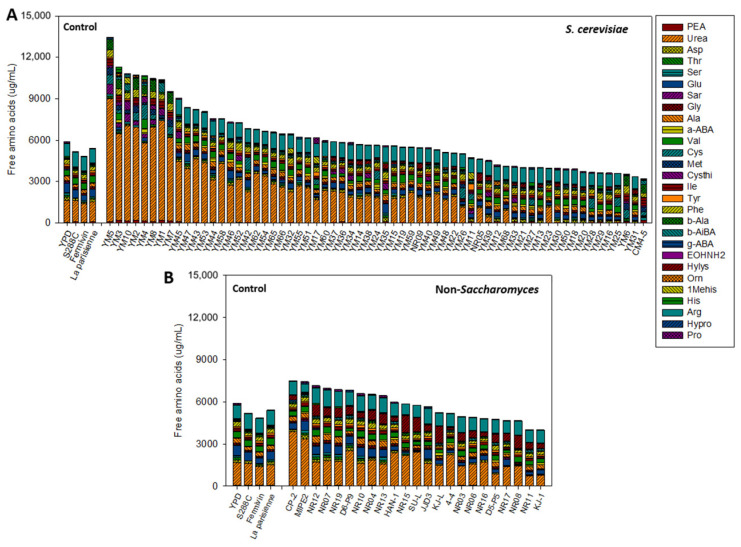
Free amino acid content in fermented media produced by different yeast strains and control yeasts: (**A**) *S. cerevisiae* strains and (**B**) non-*Saccharomyces* strains. Abbreviations: PEA, O-phosphoethanolamine; Asp, aspartic acid; Thr, threonine; Ser, serine; Glu, glutamic acid; Sar, sarcosine; Gly, glycine; Ala, alanine; α-ABA, α-Amino-n-Butyric acid; Val, valine; Cys, cysteine; Met, methionine; Cysthi, cystathionine; Ile, isoleucine; Tyr, tyrosine; Phe, phenylalanine; β-Ala, β-alanine; β-AiBA, β-aminoisobutyric acid; γ-ABA, γ-aminobutyric acid; EOHNH_2_, ethanolamine; Hylys, hydroxylysine; Orn, ornithine; 1Mehis, 1-methylhistidine; His, histidine; Arg, arginine; Hypro, hydroxyproline; pro, Proline. Urea was included as a detected nitrogen-containing compound and was not classified as a free amino acid.

The free amino acid contents are shown in Figure 5. A total of 28 free amino acid-related and nitrogen-containing compounds, including urea, were detected. *S. cerevisiae* strains showed significantly higher total levels of detected free amino acid-related and nitrogen-containing compounds than non-*Saccharomyces* strains (*p* < 0.05). The predominant amino acids in *S. cerevisiae* were urea, arginine, phenylalanine, and valine, whereas non-*Saccharomyces* strains showed relatively higher levels of urea, arginine, hydroxylysine, and glutamic acid. Total amino acid contents ranged from 3222.43 to 13,471.86 μg/mL in *S. cerevisiae* and from 4000.11 to 7509.85 μg/mL in non-*Saccharomyces* strains. *S. cerevisiae* YM5 showed the highest total amino acid content. YM17 showed the highest levels of sweet-tasting amino acids, whereas YM35 showed the highest glutamic acid content. Among non-*Saccharomyces* strains, CP-2 showed the highest total amino acid content. *S. fibuligera* NR12 showed the highest levels of sweet-tasting amino acids, whereas *Z. rouxii* JJD3 showed the highest glutamic acid content.

## 4. Discussion

This study revealed clear differences in fermentation characteristics between *S. cerevisiae* and non-*Saccharomyces* yeast strains isolated from traditional Korean fermented foods. Non-*Saccharomyces* strains were characterized by higher levels of selected esters compounds, particularly ethyl acetate and phenylethyl acetate, which have been reported to be associated with fruity and floral odor characteristics. These compounds are generally considered aroma-active molecules because of their low sensory thresholds, although their actual sensory impact depends on concentration, matrix composition, and product type [15,16]. The high ester production observed in strains such as *W. anomalus*, *C. fabianii*, and *K. marxianus* is consistent with previous studies reporting that non-*Saccharomyces* yeasts contribute to enhanced aroma complexity in fermented beverages [17,18]. In particular, the elevated ethyl acetate production observed in *W. anomalus* strains may be associated with increased acetic acid levels, as previously reported in soju fermentation [19]. Phenylethyl acetate, formed through esterification of phenylethyl alcohol with acetic acid, contributes honey-like and rose-like aromas [20,21], and its high production in non-*Saccharomyces* strains further supports their potential to enhance floral aroma characteristics. In contrast, *S. cerevisiae* strains produced higher levels of higher alcohols, including isoamyl alcohol and phenylethyl alcohol, which are formed via the Ehrlich pathway from amino acids such as leucine and phenylalanine [22]. While moderate concentrations of these compounds contribute positively to aroma complexity, excessive accumulation may result in undesirable sensory properties [23,24,25]. Interestingly, *K. marxianus* KJ-L exhibited high production of both esters and higher alcohols, suggesting that certain non-*Saccharomyces* strains may combine desirable aroma traits typically associated with both yeast groups. The non-*Saccharomyces* strains were compared as a group to evaluate overall differences from *S. cerevisiae* strains, while species-specific conclusions were limited for species represented by only one or a few strains.

Organic acid profiles also differed markedly between the two yeast groups. *S. cerevisiae* strains showed higher production of succinic acid, whereas non-*Saccharomyces* strains exhibited higher levels of acetic acid and lactic acid. Succinic acid is known to contribute salty, bitter, and acidic taste attributes and is typically produced at higher levels in *S. cerevisiae* [26]. Therefore, the relatively lower production of succinic acid in non-*Saccharomyces* strains may be advantageous for reducing bitterness in fermented beverages. The elevated acetic acid production observed in *W. anomalus* strains is consistent with previous reports indicating that increasing the proportion of this yeast during fermentation leads to higher acetic acid levels [19]. Because acetic acid is a volatile compound that can negatively affect sensory quality at high concentrations, its production must be carefully controlled. In contrast, lactic acid production was significantly higher in certain non-*Saccharomyces* strains, particularly *P. deserticola* MIPE2. This result aligns with previous studies reporting that most *S. cerevisiae* strains lack efficient lactic acid production pathways, whereas some non-*Saccharomyces* species can produce substantial amounts [27], and lactic acid contributes to acidity, microbial stability, and overall flavor balance in fermented beverages such as makgeolli. Malic and citric acids also exhibited strain-dependent variation. Although most yeasts produce higher levels of succinic acid than malic acid, the relatively high malic acid production observed in *S. cerevisiae* YM60 suggests potential for improving freshness and acidity [28,29,30,31,32]. In addition, organic acids such as malic, citric, succinic, and lactic acids contribute to microbial stability and may promote ester formation during fermentation and distillation [33]. In addition, previous studies have reported that organic acid levels and related physicochemical properties can change dynamically during fermentation, depending on microbial activity and fermentation progress [34,35,36].

Free amino acids also played an important role in distinguishing the metabolic characteristics of the yeast strains. *S. cerevisiae* strains exhibited higher total free amino acid contents, reflecting more active nitrogen metabolism. Amino acids not only influence taste [37,38] but also serve as precursors for aroma compounds via the Ehrlich pathway [22]. The higher amino acid content in *S. cerevisiae* may explain the increased production of higher alcohols observed in this study. However, excessive levels of certain amino acids, particularly sulfur-containing amino acids such as methionine and cysteine, may lead to the formation of undesirable sulfur compounds, resulting in off-flavors such as “yeast-like” or “musty” odors [33]. In contrast, non-*Saccharomyces* strains exhibited relatively lower levels of these compounds, suggesting a reduced risk of off-flavor formation. Additionally, the presence of taste-related amino acids such as glutamic acid and arginine indicates their potential contribution to improved taste balance. Because YPD contains yeast extract and peptone, these profiles may reflect uptake, release, transformation, and residual medium components rather than direct amino acid production alone.

One limitation of this study is its use of standardized YPD culture conditions with endpoint analysis after 24 h. Therefore, the present results should be interpreted as comparative screening profiles of strain-dependent metabolites within the tested culture system. Further validation in rice-based fermentation systems, together with fermentation kinetics and sensory-based analyses, will help clarify the practical flavor-related properties of the selected strains. In addition, genetic or pathway-level analyses would help explain the metabolic differences observed among candidate strains in future studies.

Overall, these results demonstrate that *S. cerevisiae* and non-*Saccharomyces* strains possess complementary fermentation characteristics. While *S. cerevisiae* contributes strong fermentative capacity and higher alcohol production, non-*Saccharomyces* strains enhance ester formation, acidity, and flavor complexity. Therefore, mixed or sequential fermentation using selected strains may be considered as a future approach to diversify flavor-related metabolite profiles in fermented beverages, although validation in actual fermentation systems is required.

## 5. Conclusions

This study evaluated the fermentation-related metabolite profiles of 63 *S. cerevisiae* and 23 non-*Saccharomyces* strains isolated from traditional Korean fermented foods. Under standardized YPD culture conditions, the two yeast groups showed distinct patterns in volatile compounds, organic acids, and free amino acid-related compounds. Non-*Saccharomyces* strains were characterized by relatively higher ester levels, whereas *S. cerevisiae* strains generally showed higher alcohol and free amino acid-related compound levels. These differences indicate that indigenous *S. cerevisiae* strains and non-*Saccharomyces* strains possess distinct and potentially complementary metabolic traits. Overall, these findings provide a useful basis for selecting candidate indigenous yeast strains with distinct and complementary flavor-related metabolic traits. Validation in rice-based fermentation systems will further clarify their practical value for fermented food development.

## Figures and Tables

**Table 1:** Yeast strains used in this study.

Yeast Group	Species	Strain Name	Number of Strains	Isolation Sources
*Saccharomyces*	*Saccharomyces cerevisiae*	CM4-5, YM1~YM68, NR05, NR09	63	Makgeolli
Non-*Saccharomyces*	*Wickerhamomyces anomalus*	KJ-1, CP-2, HAN-1, NR06, NR07, NR11, NR16	7	Nuruk
	*Saccharomycopsis fibuligera*	4-4, NR10, NR12, NR13, NR19	5	Nuruk, rice straw, malt
	*Pichia deserticola*	MIPE2, NR03	2	Fermented broth
	*Candida sorbosivorans*	D6-P9	1	Doenjang
	*Candida glabrata*	NR08	1	Nuruk
	*Clavispora lusitaniae*	NR15	1	Nuruk
	*Cyberlindnera fabianii*	NR17	1	Nuruk
	*Debaryomyces hansenii*	D5-P5	1	Doenjang
	*Hyphopichia burtonii*	NR04	1	Nuruk
	*Kluyveromyces marxianus*	KJ-L	1	Nuruk
	*Meyerozyma guilliermondii*	SU-L	1	Nuruk
	*Zygosaccharomyces rouxii*	JJD3	1	Doenjang

## Data Availability

The original contributions presented in this study are included in the article. Further inquiries can be directed to the corresponding author.
